# Avoidance of causality outside experiments: Hypotheses from cognitive dissonance reduction

**DOI:** 10.1177/00368504241235505

**Published:** 2024-04-03

**Authors:** Michael Höfler, Alexander Giesche

**Affiliations:** 1 Clinical Psychology and Behavioural Neuroscience, Institute of Clinical Psychology and Psychotherapy, 9169 Technische Universität Dresden, Dresden, Germany

**Keywords:** Causality, causal effects, non-experimental studies, avoidance, cognitive dissonance, teaching

## Abstract

The avoidance of causality in the design, analysis and interpretation of non-experimental studies has often been criticised as an untenable scientific stance, because theories are based on causal relations (and not associations) and a rich set of methodological tools for causal analysis has been developed in recent decades. Psychology researchers (n = 106 with complete data) participated in an online study presenting a causal statement about the results of a fictitious paper on the potential effect of drinking clear water for years on the risk of dementia. Two randomised groups of participants were then asked to reflect on the conflict between the goal of approaching a causal answer and the prevailing norm of avoiding doing so. One of the two groups was also instructed to think about possible benefits of addressing causality. Both groups then responded to a list of 19 items about attitudes to causal questions in science. A control group did this without reflecting on conflict or benefits. Free-text assessments were also collected during reflection, giving some indication of how and why causality is avoided. We condense the exploratory findings of this study into five new hypotheses about the how and why, filtered through what can be explained by *cognitive dissonance reduction theory*. These concern the cost of addressing causality, the variety of ways in which dissonance can be reduced, the need for profound intervention through teaching and social aspects. Predictions are derived from the hypotheses for confirmation trials in future studies and recommendations for teaching causality. Open data are provided for researchers’ own analyses.

## Introduction

### The avoidance of causality outside experiments

Causality should not be avoided in study design, analysis and interpretation of results. This is true whether or not an experimental study is possible. To understand why avoidance occurs, cognitive dissonance reduction seems a promising starting point. The problem arises because many factors of interest, especially in life sciences such as Psychology, cannot be manipulated for practical or ethical reasons.^1–3^ However, it is essential to investigate their effects on outcomes since theories are based on causal, not associational, relations. For example, scientists want to understand the aetiology of mental disorders and assess the potential impact of interventions. While the term 'causal epidemiology' has long been used to refer to the aetiological susceptibility-stress model,^4^ almost only associational analyses have been conducted, which has been criticised.^5^ Addressing the causal objective is often evaded in Psychology and other disciplines by invoking the mantra ‘correlation is not causation’,^2^ a stance that has been refuted for ‘conflating the means [no experiment is possible] with the ends [causal assessment is not required]’.^1^ The prevailing advice to circumvent causality when no experiment is possible has been condemned as detrimental to science.^1,2^

Avoidance misses the potential of the extensive methods developed over the past 40 years for designing and analysing causal effects in observational studies.^6–8^ These methods make assumptions that go beyond the data and address the specific problem of causal inference: common causes of a factor and an outcome. It has been argued that careful application of the new methods is likely to produce results that are closer to causal relations than associations, and that using them makes their assumptions transparent (e.g. ‘we assume that there are no other common causes than the variables we have adjusted for’).^3,9–11^ Despite several calls for these now not entirely new causal methods,^3,12,13^ the field of Psychology in which the authors of this article work has proved largely resistant to this need.^3,5,12,13^

At the same time, the causal interpretation of associations appears to be widespread, as suggested by the large evidence for the conflation of causal and associative language.^9,14–21^ Taking associations as causal appears necessary to give them otherwise lacking substantive meaning,^17^ but this relies on intransparent and undefendable assumptions such as that factor and result have no common causes at all.^3,13,22,23^ As Grosz et al.^17^ explain, circumventing explicit causality serves (intentionally or unintentionally) to convey a causal message because the use of explicit causal language creates salience for methodological caveats. Thus, avoidance facilitates the communication of a causal conclusion. Indeed, when an abstract does not use explicit causal language, recipients have been found to interpret the results more often causally.^24^ However, when challenged methodologically, researchers often defend their findings as merely reflecting associations.^17^

### Cognitive dissonance reduction

Grosz et al.^17^ do not explicitly refer to cognitive dissonance, but their explanations seem to draw on it. *Cognitive dissonance reduction theory*^25–27^ states that ‘when people have two thoughts that are psychologically inconsistent, they experience an aversive arousal that motivates them to change one of the cognitions’.^26^ Applied to avoidance of causality, we suggest that researchers primarily down-weight the *dissonant cognition*: the need for causal assessment and up-weight the *generative cognition* (which, according to Beauvois and Joule^25^ is) ‘the cognition most resistant to change’. Here it is the ‘norm’ that declares causality ‘taboo’ outside of experiments.^17^ We assume that this cognition usually dominates. Matters change, however, once researchers have to inform theories and justify their associational analyses. Here, the dissonant cognition demands that theory be fed with associational results (a statistically significant association appears to confirm the existence of a causal effect), but causality must remain implicit to maintain the generative cognition. This may explain the findings on blending of association and causation in research papers. We further posit that the prevailing dominance of the generative cognition, challenged but not overcome by the dissonant cognition, constitutes a ‘psychological process’, that is, a recurrent pattern that can be induced in an experiment and plays a role in long-term behaviour,^26^ in the design, conduct and interpretation of studies. In research practice, the dissonant cognition can only be observed indirectly, for example in the blending of causal and associational language when interpreting results.

According to Festinger's original formulation of the theory,^27^ conflicting cognitions lead to psychological discomfort, which is addressed by avoiding dissonance or changing cognition, attitudes or behaviour (e.g. advocating the dispensability of causality). Thus, we use the term ‘avoiding causality’ in the broad sense of not addressing causality through evading dissonance or changing cognition, attitudes or behaviour. We assume that this serves to ‘reduce the aversive feeling and restore consonance’,^27^ in the sense of a ‘recurrent psychological process’^26^ that manifests itself in research practice in design, analysis and (to a lesser extent) evaluation of results (which may suggest causality e.g. if a large association is found, assuming e.g. accurate measurement and little selection bias). In our broad notion, avoidance also includes safety behaviour, which may manifest itself by researchers turning to what is known and generally accepted, here that causality can allegedly only be assessed with the ‘gold standard’ of randomised controlled trials (RCTs) and otherwise ‘retreating into the associational haven’.^28^

We regard cognitive dissonance reduction theory as a promising starting point for explaining why researchers circumvent causality, as it has been already applied to explain obstacles against methodical and statistical conceptions.^29,30^ Moreover, it is a powerful account of how divergent cognitions are dealt with, while also being compatible with the contemporary view of the important role of emotion regulation in conflict situations.^31^

In this article, we report an online study that sought to assess how making explicit the conflict between causal avoidance and causal necessity affects motivation to address causality, and what responses to attitudes on causal questions are elicited when this conflict (and thus assumed cognitive dissonance) is induced in an experiment. From the explorative findings, we propose a set of hypotheses, which we adopt and adapt from a recent review on reducing cognitive dissonance.^31^ These hypotheses are formulated in broad terms to invite studies specifically designed to refine them. With one exception (social aspects of avoidance), we restrict ourselves to hypotheses that seem promising on the basis of our findings. Recommendations for further studies and for teaching causal methods are derived.

## Methods

### Study concept and sampling

We conducted a worldwide online experiment and collected data from 7 March to 26 April 2022. The aim of the study was to assess the attitudes of Psychology researchers towards causality and to induce a conflict between the necessity to address causality and the taboo of not doing so. An example of an associational study with a causal conclusion was presented. This was intended to trigger a psychological process in participants of ‘experiencing an aversive arousal that motivates them to change one of the cognitions’.^26^ Explication of the conflict in two of the three experimental groups was expected to enhance this process by activating the otherwise inactive necessity cognition. We hypothesised that explication would reduce the outcome *motivation to address causality*. Cognitive dissonance reduction was assumed to be the ‘mediating psychological process’, that is, the explanation for the effect, if it exists.^26^ All three groups completed a list of 19 previously unvalidated items. These were pilot tested with 10 psychological researchers and aimed to assess motivation and attitudes towards causality. The participants were suggested to read Rohrer^13^ and asked if they would be willing to participate in a follow-up assessment 4 weeks later. Only 27 out of 106 (9 from each group) did so, answering three knowledge questions (rated *no*, *yes*, *don't know*) that they should have been able to answer correctly if they had read Rohrer^13^ and thus showing motivation to engage with the new methods. Due to the sparse data, the results are not reported.

Researchers from all fields of Psychology who had published in any Psychology journal in 2020 were included in the sampling procedure. We did this to represent the discipline as a whole. (Researchers from other disciplines were thereby excluded from the outset.) Participants were sampled by journal. From a total of 1314 Psychology journals 3909 email addresses of corresponding authors were randomly selected. The first 500 were used for the invitation to participate on 7 March 2022. By 22 March, only 12 had completed the study. We then decided to contact all the remaining 3409 collected email addresses and each a second time, if necessary, to increase the response rate. On the 18th of April, there were 92 complete and 449 incomplete assessments. We closed the survey on 26 April.

### Sample size calculation

The required sample size was calculated according to the pre-registered hypotheses of the group effects on motivation, which are detailed below. With a one-tailed *t*-test, α = .05, power = 0.95 and an expected difference of 0.66 between two compared groups on the Cohen's d scale, n = 51 participants per group was planned as required (calculated using Stata, 15.1^32^ and the *power twomeans* command). This applies to both of two hypotheses and gives a total sample size of 153, with equal probability of assignment to the three groups. In terms of the precision of the group effect estimates, the group size of 51 yields an expected 95% confidence interval (CI) width of 0.78 (independent of the effect estimate, command *ttesti*).

### Statistical analyses

Linear regressions with robust standard errors, two-tailed tests and 95% CIs were used to examine group differences in item ratings and to test the pre-registered hypotheses in an exploratory manner (with a post hoc weakened inclusion criterion for this). Rank correlations with two-tailed p-values and 95% CIs were calculated for associations between items. All analyses were conducted with Stata, version 15.1.^32^ Open data are also provided in R format.

We give more details of the experimental procedure in the results chapter, where it is easier to put the findings into context. Further information on the pre-registered hypotheses and sampling details is provided in the Appendix. The OSF project page (https://osf.io/msn9r/) adds materials, all items used (with variable names and codes) and open data for reproducibility and own analysis. It also includes all analyses carried out by the authors and the history record of hypotheses, analysis plans and sampling. The study was conducted with Limesurvey.^33^

## Results

### Participation

**Figure 1.**
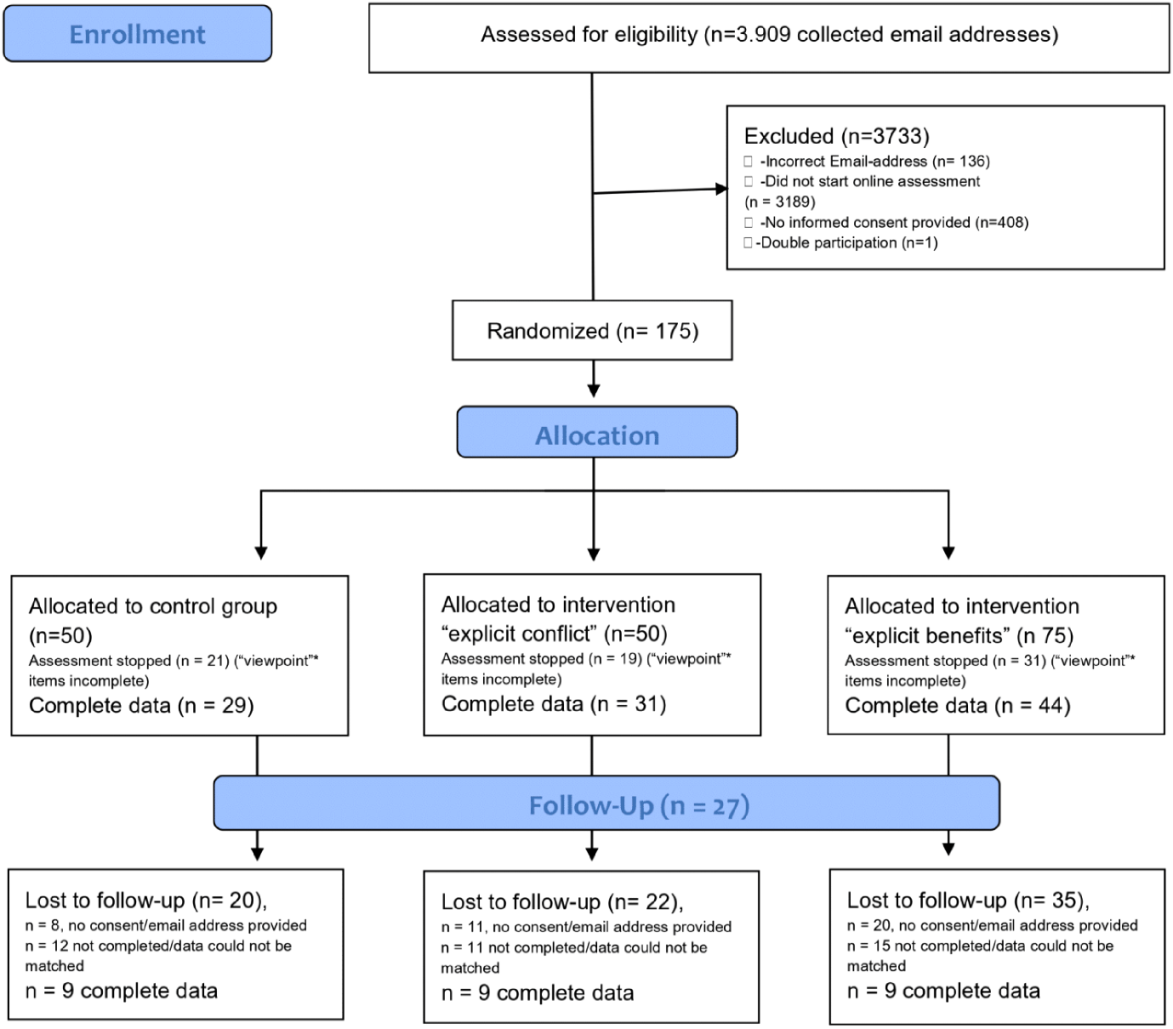
Consort flow diagram of the online study conducted.

One hundred and seventy-five Psychology researchers (106 with complete data) participated in the study. It was titled *Study on approaches to scientific questions* (we quote from the study protocol in italics), which was intended to be neutral in the sense that it did not activate the cognitions under consideration from the outset. Figure 1 shows the consort flow diagram. We did not apply any exclusion criteria as all participants were part of the target population (Psychology researchers) through sampling and we were interested in exploring all participants’ responses to the study.

Table 1 describes the socio-demographic distribution in the sample.

**Table 1.** Socio-demographic distribution among n = 106 participants.

		Total		Control group		Conflict group		Benefits group	
	.	(n = 106)		(n = 30)		(n = 31)		(n = 45)	
Academical status		**n**	**%**	**n**	**%**	**n**	**%**	**n**	**%**
	Undergraduate student	1	0.9	0	0.0	1	3.2	0	0.0
	PhD student	9	8.5	2	6.7	2	6.5	5	11.1
	Postdoctoral student	10	9.4	3	10.0	2	6.5	5	11.1
	Faculty	69	65.1	19	63.3	20	64.5	30	66.7
	Other	17	16.0	6	20.0	6	19.4	5	11.1
Research discipline in psychology									
	Clinical	24	22.6	9	30.0	5	16.1	10	22.2
	Cognitive	9	8.5	1	3.3	2	6.5	6	13.3
	Neuroscience	7	6.6	1	3.3	4	12.9	2	4.4
	Developmental	10	9.4	3	10.0	1	3.2	6	13.3
	Quantitative	9	8.5	3	10.0	3	9.7	3	6.7
	Social/personality	13	12.3	2	6.7	6	19.4	5	11.1
	Other	34	32.1	11	36.7	10	32.3	13	28.9
Subfield									
	Clinical	26	24.5	12	40.0	6	19.4	8	17.8
	Cognitive	12	11.3	0	0.0	3	9.7	9	20.0
	Neuroscience	8	7.6	0	0.0	4	12.9	4	8.9
	Developmental	7	6.6	3	10.0	0	0.0	4	8.9
	Quantitative	11	10.4	3	10.0	4	12.9	4	8.9
	Social/personality	16	15.1	3	10.0	8	25.8	5	11.1
	Other	26	24.5	9	30.0	6	19.4	11	24.4
Age	.								
	18–25	2	1.9	1	3.3	1	3.2	0	0.0
	25–35	25	23.6	8	26.7	10	32.3	7	15.6
	35–45	34	32.1	9	30.0	9	29.0	16	35.6
	45–55	22	20.8	8	26.7	6	19.4	8	17.8
	55–65	11	10.4	1	3.3	3	9.7	7	15.6
	66+	12	11.3	3	10.0	2	6.5	7	15.6
Gender									
	Male	53	50.5	16	55.2	14	45.2	23	51.1
	Female	51	48.6	12	41.4	17	54.8	22	48.9
	Other	1	1.0	1	3.5	0	0.0	0	0.0
	The United States	27	26.0	6	20.0	6	20.0	15	34.1
	Great Britain	7	6.7	0	0.0	1	3.3	6	13.6
	European Union	39	37.5	11	36.7	14	46.7	14	31.8
	Other	31	29.8	13	43.3	9	30.0	9	20.5
How old when learning English?									
	Native speaker	36	34.0	8	26.7	5	16.1	23	51.1
	Before age of 10	30	28.3	11	36.7	11	35.5	8	17.8
	… between 11 and 19	35	33.0	9	30.0	14	45.2	12	26.7
	… when aged 20 or older	5	4.7	2	6.7	1	3.2	2	4.4

### Results of the experimental part

After answering socio-demographic questions, the participants were presented with the following text:

*A recent study investigated whether drinking one litre of clear water per day would reduce the risk of dementia in late life. The study managed to look at 100,000 individuals longitudinally over the lifespan and came up with a statistically significant and stable estimate (neglectably small standard error) of a 50% risk reduction for dementia in persons who drank at least one litre of clear water (for at least 10 years). The result was adjusted for age and sex.*

*Then the nutrition researcher concludes: 'Based on this result, I consider drinking a litre of plain water each day, because I can expect that this would reduce my dementia risk by 50%.'*

This example seems valid because the effect of drinking one litre of clear water over a period of 10 years or more cannot be studied under laboratory conditions, and humans cannot be forced (not) to drink that much water for that long. Only an observational study is possible here, and the specific problem of causal investigation applies: the decision to drink a lot of clear water for a long time and the outcome dementia are likely to be influenced by several common causes including somatic conditions and health behaviours.^34^

Two manipulation check items followed: *the example describes a (fictional) dialogue between two scientists* and *drinking plain water over years can only be investigated in an observational study*. The latter aimed to test whether a participant understood that an RCT was impossible in this example. The participants were then randomly assigned to three groups. The *conflict group* (n = 31) and the *benefits group* (n = 44) were presented with the two dissonant statements:

- *A causal conclusion must not be made with a non-experimental study like this.*
- *A causal conclusion is unavoidable, because one must decide whether to start drinking plain water to prevent dementia*.

The taboo statement was largely confirmed with 4 respondents answering *very weakly/not at all*, 9 *weakly*, 17 *moderately*, 27 *strongly* and 40 *very strongly*, whereas the second necessity statement presented was not confirmed by most: 54 *very weakly/not at all*, 26 *weakly*, 12 *moderately*, 3 *strongly* and 2 *very strongly*. Both groups were then asked *do you believe that the last two statements are in conflict?* and were instructed to *write down your thoughts*. They were given exactly one minute to do this. Only the benefits group were posed a furtherquestion: *Do you believe that research could move forward if it addressed causality in non-experimental instances like this? (using sound methods like adjusting for common causes of factor and outcome)*. Again, they were given one minute to write down their thoughts on this. We left only one minute to avoid the risk of participants dropping out due to the length of time it took to process, but we later realised from some email responses that a longer time might have been better.

All three groups completed a list of 19 items on attitudes towards and barriers to causal inquiry, the *control group* (n = 29), without being presented with the two statements above and without thinking about conflict and benefits. All items used the aforementioned 5-point Likert scale from 1 = *strongly disagree* to 5 = *strongly agree*.

### Pre-registered hypotheses

The last 2 of the 19 attitude items asked about the motivation to address causality in non-experimental studies:

*I feel motivated to address causality outside experiments in my field.*

*I feel motivated to dig into methods if necessary for this purpose.*

The mean of these two defined the outcome of the following pre-registered hypotheses:

1. Motivation is reduced after reflecting about the conflict
2. Motivation is higher when also reflecting about benefits of addressing causality

Confirmatory tests of the hypotheses could not be conducted because only 8 out of 106 participants with available outcome information met the pre-registered inclusion criteria for the analysis. These had been defined by the manipulation check items, requiring (a) confirmation that *the example describes a (fictional) dialogue between two* scientists and (b) *strongly* or *very strongly* agreeing with *drinking plain water over years can only be investigated in an observational study*. Most were lost by (b), only 11 out of 106 answered as expected: 30 *very weakly/not at all*, 38 *weakly*, 27 *moderately*, 10 *strongly* and 1 *very strongly.* In total, without excluding those who later dropped out, 138 responded to these items, 40 endorsed *very weakly/not at all*, 50 *weakly*, 33 *moderately*, 14 *strongly* and 1 *very strongly*.

Thus, a major finding of our study is that the pre-registered analysis did not work. Nevertheless, we examine the hypotheses with a weaker inclusion criterion below. Together with an exploration of the data on attitudes to causal questions, this leads to five newly proposed hypotheses.

## Suggested hypotheses on causal avoidance

The following cognitive dissonance reduction explanations appear useful for *understanding* causal avoidance and for *intervention* with teaching students and researchers. Firstly, we would like to emphasise that all suggested explanations, which are taken and adapted from Cancino-Montecinos et al.,^31^ are *post hoc*. When planning the study, we had a rather vague idea of how cognitive dissonance reduction might explain avoidance of causality. The plan to condense the exploratory findings into a set of *newly proposed hypotheses* came only later. New data are required to test these hypotheses or the predictions they make, and the next section makes suggestions for this. We restrict ourselves to only a few hypotheses that are supported by initial evidence and seem promising to be confirmed later.^35,36^ The analyses reported in the following have been stored there.

### Was causal avoidance actually triggered by the example in this study?

The following hypotheses depend heavily on whether our study design succeeded in inducing a psychological process of causal avoidance. First, such a process may have already been triggered by the second manipulation check item. We did not expect an objection yet to the statement *drinking plain water over years can only be investigated in an observational study*. The disagreement suggests the belief that an experimental study is possible, perhaps with an animal study. This may indicate an attempt to avoid the issue in general or a particular safety behaviour to maintain the generative cognition by reaching for the gold standard RCT, which is actually not available.

A second piece of evidence is the response frequencies to the two conflicting statements mentioned above, which suggest widespread agreement with the taboo and widespread disagreement with the necessity. Also, several frequencies of responses to the 19 attitude items seem unlikely if avoidance was rare, as shown in Table 2.

**Table 2.** Results on 19 attitude items in n = 106 participants.

Control group		Conflict group		Benefits group		Conflict vs. control group	Benefits vs. control group
(n = 30)		(n = 31)		(n = 45)			
Mean	SD	Mean	SD	Mean	SD	p*	p*
3.6	1.2	3.8	1.0	3.4	1.2	0.684	0.517
2.8	1.2	2.4	1.0	2.2	1.1	0.209	0.057
2.8	1.4	3.0	1.2	2.6	1.2	0.487	0.620
3.1	1.2	2.7	1.0	3.0	1.2	0.217	0.938
3.3	1.3	3.4	1.2	2.9	1.3	0.868	0.144
2.7	1.4	2.6	1.3	2.5	1.4	0.949	0.637
3.2	1.1	3.2	1.0	3.2	1.2	0.978	0.840
3.3	1.0	3.3	0.8	3.0	0.8	0.921	0.287
2.0	1.0	2.1	1.0	2.0	1.2	0.613	0.758
1.7	1.1	1.6	0.7	1.4	0.9	0.707	0.273
1.8	0.9	1.6	1.0	1.5	0.9	0.600	0.157
3.9	1.1	3.5	1.2	3.9	1.1	0.235	0.838
2.6	1.1	2.5	1.2	2.4	1.3	0.777	0.610
2.3	1.1	2.7	1.0	2.2	1.3	0.091	0.756
2.7	1.2	2.3	0.9	2.0	1.2	0.178	**0**.**030**
3.1	1.2	3.0	1.0	2.9	1.2	0.811	0.439
2.3	1.1	2.2	1.0	2.0	1.0	0.882	0.224
3.2	1.1	2.6	1.1	2.6	1.2	0.067	**0**.**032**
4.0	1.0	3.6	1.1	3.6	1.1	0.180	0.182
* two-tailed p-value from linear regression with robust standard error							
** List of items:							
1. Causal conclusions must only be made with experimental studies (randomised experiments or randomised clinical trials, RCTs).							
2. I feel insecure in dealing with causality.							
3. I fear to make wrong causal claims, therefore I only investigate associations if an experiment is impossible.							
4. In my own field investigating associations covers the core research questions.							
5. Everything that is necessary for a causal conclusion must be taken from the data.							
6. Conducting an experimental study protects against bias through a selective, non-representative sample.							
7. Conducting an experimental study protects against bias through common causes of a factor of interest and an outcome (confounding bias).							
8. When adjusting for confounders I use the same confounders than other authors in my field do.							
9. Causality is ultimately a matter of belief.							
10. A researcher’s belief justifies a causal conclusion.							
11. A correlation most often indicates a causal effect.							
12. It is hard to address each and every aspect in a study including causality.							
13. I prefer to forward methodical issues like causality to a methodical expert/statistician.							
14. Avoiding explicit causal language (‘causal effect’) in a paper on a non-experimental study makes readers more likely to draw a causal conclusion.							
15. Causality is ultimately a matter of definition.							
16. It is sufficient to mention the limitation that a causal conclusion cannot be drawn.							
17. I fear that adjustment for confounders would let my finding of an effect diminish. ´							
18. I feel motivated to address causality outside experiments in my field.							
19. I feel motivated to dig into methods if necessary for this purpose.							

Further indication of avoidance can be taken from some of the results below, and readers are invited to evaluate the avoidance assumption against their own analyses.

### Does cognitive dissonance reduction indeed occur under exposure to the conflict?

The second pre-requisite for the following hypotheses to be supported by the results of the study is that dissonance reduction was actually induced by our example and no other psychological processes were triggered. The first indication of this are the results on the hypotheses when the inclusion criteria were weakened to require only a *yes* response to *the example describes a (fictitious) dialogue between two scientists*. Among the 85 participants now included, those in the control group (n = 25, mean = 3.6, SD = 0.8) showed the highest motivation in the sample, while motivation was lower in the conflict group (n = 26, mean = 3.1, SD = 0.9, difference as compared to controls = −0.46, 95% CI = −0.94 to 0.02) and in the benefits group (n = 34, mean = 3.1, SD = 0.9; difference as compared to controls = −0.49, CI = −0.94 to −0.03). (The CIs suggest a true difference of negligibly small size up to a difference of almost one standard deviation).

Second, we evaluated and categorised the responses to the free text item when reflecting on the conflict (total n = 91). No particular qualitative method was used for this, but categorisation was done until both authors agreed. The free text responses are available there (https://osf.io/ynu2e) for the readers to examine for themselves (e.g. with thematic analysis), and perhaps to refute our interpretations on them. Forty-five participants denied the conflict (at least as a scientific conflict), 5 did not comment on the conflict (of which 2 only called for caution which is scientifically adequate), 3 gave scientifically inadequate answers, 27 confirmed the conflict and 3 gave an otherwise positive comment. Eleven reported technical problems or that the task was unclear or gave an incomplete answer. A total of 48 (45 + 3, 53%) showed a potential effect of dissonance reduction (95% CI = 42%–63%).

Finally, pooled across the *conflict* and the *benefits groups*, we found a negative association (rank correlation = −0.27, 95% CI = −0.53 to −0.12) between the conflicting items *a causal conclusion is unavoidable, because one must decide whether to start drinking plain water to prevent dementia* and *causal conclusions must only be made with experimental studies*. Thus, the affirmation of the taboo may go along with the denial of the inevitability.

However, we cannot rule out that participants in the *conflict* and the *benefits groups* reported less motivation simply because of the alternative psychological process of annoyance (some complained in emails about technical problems and about having only one minute to think).

### Hypothesis 1: Avoiding causality is rewarded because addressing causality has large costs

Cognitive dissonance theory suggests that ‘people make more or less conscious cost-benefit analyses when deciding how to regulate emotions’, and between conflicting long-term and short-term goals.^31^ A long-term goal in science is to achieve profound and sustainable results, while short-term goals focus on career opportunities through rapid publication. Causal relationships provide answers to more sophisticated questions than associations do, but their analysis requires much more effort: digging into methods, thinking about the mechanisms behind the data, and justifying explicit assumptions and the use of the unfamiliar analyses, which invite objections requiring further action.

This argument seems to concern only those scientists who are aware of the possibility of causal analysis and how it largely works, but others may suspect that causal analysis is cognitively demanding anyway. In contrast to this effort, avoidance seems to allow the data to ‘speak for themselves’ in the form of associations, creating an illusory objectivity.^37^ Furthermore, adherence to the social norm of respecting the ‘taboo against causal inference’^17^ facilitates publication and protects the integrity of long-standing practices and one's own integrity as a researcher. Thus, the costs of addressing appear to far outweigh the benefits.

In addition to these incentive arguments, the results of Alvarez-Vargas et al.^24^ suggest that refraining from causal explanation is even rewarded with results that are perceived as more methodologically sound. Our study provides at least some indirect evidence through the rank correlations between the acceptance of inevitability and the attitude items, three of which (items 2, 12 and 17) relate to the costs of dealing with causality. These correlations are shown in the last column of Table 3. They suggest that acceptance of unavoidability comes at the expense of the emotional cost of feeling *insecure in dealing with causality* (item 2, rho = 0.34, 95% CI = 0.12–0.52) and the practical cost of *fear that adjustment for confounders would let my finding of an effect diminish* (item 17, rho = 0.33, 95% CI = 0.11–0.52). However, no association was found with *it is hard to address each and every aspect in a study including causality* (item 12, rho = −0.03, 95% CI = −0.25 to 0.20).

**Table 3.** Rank correlations between three initially asked items and the 19 stance items.

… with stance	Manipulation check Item 2*			Conflicting Item 1**			Conflicting Item 2***		
Item	n	Rho	p	n	Rho	p	n	Rho	p
1	107	−0.05	0.601	77	0.45	0.000	77	−0.27	0.016
2	107	0.14	0.148	77	−0.22	0.057	77	0.33	0.003
3	106	0.00	0.994	76	−0.02	0.831	76	0.10	0.406
4	106	−0.06	0.553	76	−0.03	0.807	76	0.19	0.108
5	106	−0.06	0.571	76	0.14	0.245	76	0.08	0.500
6	106	0.00	0.975	76	−0.06	0.630	76	0.23	0.050
7	106	0.10	0.330	76	0.07	0.549	76	−0.03	0.764
8	106	−0.14	0.161	76	−0.12	0.288	76	−0.06	0.594
9	106	0.13	0.169	76	−0.12	0.295	76	0.24	0.037
10	106	0.13	0.186	76	−0.15	0.202	76	0.41	0.000
11	106	0.00	0.978	76	−0.36	0.001	76	0.31	0.006
12	106	−0.15	0.125	76	−0.11	0.364	76	−0.03	0.822
13	106	−0.01	0.890	76	0.01	0.931	76	0.11	0.358
14	106	0.16	0.109	76	0.04	0.762	76	0.09	0.456
15	106	0.03	0.798	76	0.04	0.740	76	0.39	0.001
16	106	−0.14	0.167	76	−0.10	0.384	76	0.03	0.796
17	106	−0.01	0.956	76	−0.05	0.671	76	0.33	0.003
18	106	0.16	0.105	76	−0.16	0.160	76	0.14	0.236
19	106	−0.02	0.845	76	−0.04	0.749	76	−0.09	0.451

*Drinking plain water over years can only be investigated in an observational study.

**A causal conclusion must not be made with a non-experimental study like this. Only asked in the conflict and the benefits group.

***A causal conclusion is unavoidable, because one must decide whether to start drinking plain water to prevent dementia. Only asked in the conflict and the benefits group.

### Hypothesis 2: Without a profound understanding of causality, the acceptance of inevitability coincides with inappropriate stances

We raised this hypothesis after finding four correlations between accepting unavoidability and agreeing with inadequate strategies for dealing with causality (Table 3, last column): *a researcher*‘*s belief justifies a causal conclusion* (item 10, rho = 0.41, 0.20–0.58), *a correlation most often indicates a causal effect* (item 11, rho = 0.31, 0.10–0.50), *causality is ultimately a matter of definition* (item 15, rho = 0.39, 0.18–0.57) and *I fear that adjustment for confounders would let my finding of an effect diminish* (item 17, rho = 0.33, 0.11–0.52).

Here, for some researchers, the cognition of need may dominate, but at the cost of addressing the need with poor means. If the statements in items 10, 11, 15 and 17 were true, no action would be required.

### Hypothesis 3: The modes of dissonance reduction are diverse

As evidence for this claim, we first refer to the full list of n = 91 free-text responses when reflecting on conflict (and benefits). Readers are invited to evaluate diversity for themselves on the basis of the unsorted and uncommented list provided here, and to come up with different classifications of the responses than we suggest there.

According to Cancino-Montecinos et al.,^31^ dissonance reduction can employ several strategies, the use of which depends on situational factors and interpersonal differences. We mention only two of these, which we believe can be identified from the free-text responses. First, researchers may ‘transcendent the conflict; i.e. seeing the big picture’.^31^ For example they might point out that scientific causality and practical recommendations are different issues. However, this contradicts what a causal effect is, as opposed to an association: a causal effect is what an intervention would do, whereas an association-based prediction is based only on passive observation of people in the factor and outcome status, which may have no relevance to action on the factor.^2,38^ (Here, the finding that drinking clear water is associated with a lower risk of dementia does not mean that one can reduce one's risk by drinking clear water, if the association is only due to common causes.) An example of transcending the problem in this way is the response:

*One can believe that a causal connection is not established in a study, yet still make decisions on the basis of non-causal evidence. One is simply making personal decisions on the basis of different criteria – e.g. probability estimates.*

(The statistical term ‘probability estimates’ is synonymous with ‘association’ and ‘prediction’ and does not imply causality).

Researchers may also choose to rationalise a prior commitment to a behaviour,^31^ for example, insisting on conducting an experimental study when this is impossible. One participant wrote:

*I think that the first one is correct and such a conclusion is ridiculous. The second one is not necessarily in conflict, but it seems to condition that one accepts the conclusion without the detailed experimental study*.

In addition, we carried out an exploratory factor analysis on the attitude items (the first 17 of them which actually involve attitudes, not motivation) in an attempt to reduce the attitudes to some potentially striking latent dimensions that might indicate low diversity. But we found no factors with a clear interpretation, also when one item was omitted. This could both indicate diversity or that our (psychometrically unexamined) items were poor. The analysis is available there.

### Hypothesis 4: Short reflection on potential benefits does not help against avoidance

As argued in Hypothesis 1, the dominance of cognitive and other costs over benefits of facing the causal question seems firmly entrenched. This suggests that a *major intervention* is required to change this imbalance, and that a brief reflection on benefits without further underpinning will not succeed. In our data and as noted above, the *benefits group* appears to be less motivated to address causality than the control group, with an estimated difference of −0.49 (half a category and about half a standard deviation, 95% CI = −0.94 to −0.03). Furthermore, in the sample, the *benefits group* was similar to the *conflict group*, with a difference of −0.02, while the confidence interval includes values up to half a standard deviation in either direction (95% CI = −0.50 to 0.49).

### Hypothesis 5: Social aspects maintain the avoidance

According to the theory social aspects play an important role in dissonance reduction: ‘the social context in which the dissonance occurs may determine the reduction strategy’, ‘the social context in which the dissonance is evoked (e.g. presence of others vs. being alone) might dictate how people reduce dissonance’.^31^ Similarly, it has been suggested that social aspects enhance conforming behaviour.^39^ Although social aspects were not assessed in our study, we raise this hypothesis because the acceptance by others is an integral part of scientific success and, as we argue in the following, the hypothesis has major implications for teaching causality as an intervention.

## Discussion

Our study has several limitations. The response rate was low, 106 participants with complete data for most analyses out of 3909 email addresses collected, or 2.7%. This may introduce considerable selection bias. In the invitation email we had announced that the study was about *how to approach scientific questions that are difficult to answer by scientific standards*. Assuming that most researchers seek confirmation rather than conflict,^40,41^ this wording should have discouraged the participation of researchers who prefer to escape difficult scientific questions. Without such selection, one would expect to find even more evidence of causal avoidance and stronger responses to its triggering, perhaps revealing more modes of avoidance. Other limitations include:

- The use of none-validated items to assess motivation and attitudes to causality.
- Priming effects, here triggering the conflict between addressing and circumventing causality, may be unreliable in between-group designs when studying a heterogeneous population.^42^
- Likewise, a within design with a pre-intervention measure would have been better able to demonstrate that a change was actually induced, internalised and relevant to the researchers themselves – pre-requisites for resolving them.
- The manipulation check item *drinking plain water over years can only be investigated in an observational study* might have been misunderstood to mean that this left open the possibility of an animal study (although *for at least ten years* in the example presented should refer to humans).
- Asking participants to read a paper may have been perceived as too demanding and may have resulted in participants not completing the follow-up assessment.

## Suggestions to test the proposed hypotheses

Some confirmatory tests of our hypotheses can be carried out with relatively simple studies.* Hypothesis 1 (avoidance is rewarded) *can be tested with items that name costs and benefits, and participants can be asked to quantify costs, benefits and their importance. The hypothesis predicts that costs (weighted by importance) will be reported as much higher.

*The diversity hypothesis (Hypothesis 3)* can be tested, refined and extended by investigating the prevalence of particular dissonance reduction strategies. A large sample can be presented with an example similar to ours, the conflict triggered (perhaps more efficiently than here), and items constructed that cover the above (and other) strategies. The hypothesis predicts that different strategies will often be chosen, so that a large variance will be found. The items in this study were formulated in rather general terms, but social psychology suggests that norms are highly context specific.^43^ Even greater variation should be found when context-specific items are used. Furthermore, participants should be given more time than one minute to reflect on the conflict, leading to more extensive free-text responses for a more in-depth qualitative assessment. Finally, qualitative interviews can provide an even fuller account of what is really going on in reducing dissonance.

*Hypothesis 4 (brief reflection does not help)* can be tested with new data using a similar example and the broader inclusion criterion applied in the exploratory analysis.

*Hypotheses 2 (in-depth intervention is needed, otherwise dealing with causality is associated with inappropriate stances) *and *5 (social aspects must be considered) *should be tested through *teaching*. Teaching should focus on causal methods and the *benefits* of considering causality (see the final paragraph for how to do this). Hypothesis 2 predicts that this will increase motivation to address causality compared to a control group taught, for example, general statistical methods. The choice of outcome seems crucial here. Simply asking about motivation might merely reflect the social desirability of those who are taught causality. Harder outcomes such as the actual use of causal methods in scientific papers are desirable but difficult to observe in a short time. The hypothesis also predicts that in-depth instruction will change the above correlations between accepting unavoidability and poor strategies for dealing with causality from positive to negative. (In the control group, the correlations should be positive again.) In addition, positive correlations should be found with adequate stances and negative correlations with uncertainty and fear in addressing causality (although the latter could be due to social desirability).

We also predict that the effects described above will be larger the less experienced the researcher. Researchers with longer careers are more likely to have a more stable generative cognition (the taboo) or more effective dissonance reduction strategies, which may render the dissonant cognition (the necessity) barely active. Finally, the hypothesis that social aspects maintain avoidance behaviour (Hypothesis 4) predicts that teaching groups will yield better results than teaching individuals.

### More focused experiments

Experiments investigating cognitive dissonance stand or fall on whether the experiment actually induces cognitive dissonance, not other psychological processes.^26^ Our study was not designed to rule out alternative processes (e.g. annoyance), nor did we use questionnaires to assess how much dissonance reduction was actually induced, internalised and relevant to the researchers. The free-text responses are too sparse to assess this accurately. Ideally, an experiment would manipulate both the factor and the mediating variable’,^26^ here conflict explication (what we did) *and* dissonance reduction. This would allow testing whether an outcome differs between groups with different levels of induced dissonance reduction (among participants who had the conflict explicated). Specifically, the ‘experimental-causal-chain design’ would run two experiments: one to test the effect of explication and one to test the effect of dissonance reduction.^26^ A valid manipulation of dissonance reduction might be to offer a solution to the explicated conflict, such as ‘remember that your chances of getting a paper published are probably higher if you don't even touch on the complicated issue of causality’. Whereas explication should activate the dissonant cognition of necessity, the induction of dissonance reduction should deactivate it again.

One can also vary the weight of the two cognitions in the provided statements to see how this changes the effect by strengthening or weakening one or the other cognitions (e.g. adding to the taboo statement: ‘Many journals only accept the results of an experimental study as causal’ or adding to the necessity statement: ‘Aetiological models of dementia require causal, not associational relations’). On top of this, the activation of the dissonant cognition requires a basic scientific knowledge to recognise the necessity of causality. Conversely, too much basic knowledge about causal methods for non-experimental studies prevents the emergence of dissonance because the taboo has been overcome. Therefore, future studies should collect information on this knowledge.

## Suggestions for teaching

### Who should be taught?

It is textbook psychological knowledge that teaching should begin with students and preferably at undergraduate level because it is easier to learn things accurately early on than to have to relearn them later. On the contrary, teaching experienced scientists must take into account that a change in behaviour challenges previous practice and the gains that have been made from it. This requires identifying strong rewards for change, such as more appropriate, deeper and more sustainable results.

### Identify benefits that are capable to change the cost–benefit balance

Teaching should first overcome the fundamental misconception that causal analysis necessarily requires an experiment. This may be achieved by teaching skills in causal analysis and the design of studies based on causal models (which enable such analysis by identifying the relevant common causes of factors and outcomes). Such knowledge should activate the necessity cognition, while reducing uncertainty and fear. Teaching should explicitly empower researchers to model the effects of interventions and to feed theories with the results of informed causal analysis rather than associations.^35^ Benefits include the prospect of improved theories,^44,45^ and thus greater sustainability of science. Empowerment in itself increases self-efficacy, and this can be enhanced by recognising that causal analysis essentially models the effects of actions, even though actions (experimental changes) are impossible: What would happen if we could change factors that we cannot actually change?^2^ In an exercise, researchers might reflect on how this would change their own self-efficacy in their current research. Another exercise could explore how causal evaluation is fundamental in everyday life by imagining counterfactual decisions and their possible outcomes.^2^ Participants could then be asked to imagine how satisfying it would be to get rid of the strange contradiction between the necessity and avoidance of causality in science. It can also be conveyed that researchers who are competent in causal methods are allowed to use clear and explicit causal language rather than vague associative language. Researchers using an explicit causal model may refer to a ‘causal effect’ and make statements such as ‘drinking clear water reduces the risk of dementia’ (this is sound if the underlying causal model holds, which must be included in the communication of this result). Similarly, scientific communication to the public will be improved because a researcher will be enabled to recommend ‘yes, it is probably worth starting to drink clear water’ rather than having to add ‘the result cannot be taken as causal’ and leaving the public with more uncertainty than necessary.

### Teach groups of researchers

To target the assumed diversity in dissonance reduction, it is important to respond to as many arguments against addressing causality as possible. The message should be that they can all be refuted. The group itself may raise obstacles, the teacher may add others, and by challenging them all in the group, common acceptance may be reached that there is no way out of the necessity of causal analysis.

A seminar group should define common goals for the group to achieve. An obvious example is the task of carrying out a causal analysis together. A dataset may be provided with several well-measured putative common causes of factor and outcome that may be adjusted for when estimating the effect of a factor on an outcome. The group's task is to construct a causal model, conduct a derived analysis, and finally come up with a causal result. Ideally, the result is easy to validate because the effect under investigation is largely known. Famously, smoking was long denied as a cause of lung cancer due to causal avoidance.^46^ The exercise can be done separately in smaller groups to see how the result depends on the choice of a causal model. Participants may then notice how a discussion about the assumptions in the model emerges naturally. They may agree that this is far preferable to the common approach of basing an analysis on assumptions that no one would defend if they were stated openly.

Another idea is to include stakeholders such as journal editors in the trained group, as they have the means to promote thoughtful causal analysis in publications, and their acceptance may facilitate adoption by researchers. Otherwise, barriers or perceived barriers from editors may remain a major obstacle.

It seems promising to train psychologists and statisticians at the same time. Some of the methods are complex and their details involve a lot of mathematics and are beyond the scope of an introductory workshop. With the statistician at their side, able to delve into the details, self-efficacy should become even greater. Also, when teams of psychologists and statisticians are trained together, the teams learn how to share the work in a joint causal investigation. This may mean that the statistician tells the psychologist what assumptions are needed and how the choice of model determines the study design and analysis, and the psychologist makes the assumptions.^47^ New tools are available to support the specification of the causal model and analysis, reducing the likelihood of errors.^22,23^

### Use illustrative examples

The use of a famous example such as the effect of smoking on lung cancer ties in with common knowledge and shows how important it was to arrive at a causal answer. Such an example may also include an illuminating history of the obstacles that have long hindered causal analysis and which have finally been overcome.^2^ Another example is the 2021 Nobel Prize in Economic Sciences, which was awarded for the development and application of recent causal methods.^48^ This example shows that a discipline has benefited from the use of causal methods, and that scientific merit can be achieved therewith.

### Address the avoidance and uncover the motivation behind it

We suggest that teaching should encourage introspection about how avoidance is motivated, what a scientist's own goals are (mainly publication and career?), and whether these might conflict with the pursuit of knowledge in science. We also propose not to impose a conclusion, but to count on the prospect of a causal result to generate motivation. Either a plausible causal model that could serve as the basis for such an analysis may immediately emerge, or it may be realised that the knowledge for such a model is too limited, so that motivation arises to first improve this knowledge to then be able to carry out a causal analysis.^3^

## Conclusion

We have put forward five hypotheses that are intended both to provide a reasonable starting point for explaining causal avoidance through dissonance reduction and to suggest how the problem might be investigated in further studies and addressed in teaching. Our hypotheses invite refinement and extension. More in-depth explanations may be drawn from cognitive dissonance reduction or other psychological theories. For reasons of scientific parsimony, we have largely confined ourselves to explanations that our study, which was not designed for this purpose, happens to cover.

We would like to conclude by stating that we are curious about anything that our paper might stimulate, any hint as to how to address and overcome the problem of causal avoidance, a goal that must not be avoided either.

## Consent

The authors confirm that all participants have provided written informed consent and have agreed to the use of their data by endorsing ‘yes’ to the statement that can be accessed there. The same applies to the data protection and privacy declaration that can be accessed there. The answers have been recorded by the LimeSurvey software.^33^ The procedure has been approved by the Dresden Ethical Committee (SR-EK-370072021). Open data, full data descriptions and materials are available in the OSF project associated with the online study.

## Supplemental Material

sj-docx-1-sci-10.1177_00368504241235505 - Supplemental material for Avoidance of causality outside experiments: Hypotheses from cognitive dissonance reduction Supplemental material, sj-docx-1-sci-10.1177_00368504241235505 for Avoidance of causality outside experiments: Hypotheses from cognitive dissonance reduction by Michael Höfler and Alexander Giesche in Science Progress

sj-pdf-2-sci-10.1177_00368504241235505 - Supplemental material for Avoidance of causality outside experiments: Hypotheses from cognitive dissonance reduction Supplemental material, sj-pdf-2-sci-10.1177_00368504241235505 for Avoidance of causality outside experiments: Hypotheses from cognitive dissonance reduction by Michael Höfler and Alexander Giesche in Science Progress

## Appendix: Methods used in the online study

This appendix adds details on the pre-registered hypotheses and the sampling procedure. The project process was continuously recorded in the OSF project. The record includes materials (the entire study process with all items used in LimeSurvey, version 5.3), Stata syntax for data processing and analysis, plans for exploratory analyses and their subsequent results, and open data.

### Pre-registered hypotheses and their analysis

#### Hypothesis 1

The conflict group shows lower motivation than the control group.

#### Hypothesis 2

The benefits group shows higher motivation than the control group. *Motivation* was defined as the mean of the items *I feel motivated to address causality outside experiments in my field* and *I feel motivated to dig into methods if necessary for this purpose*. A hypothesis was to be considered as confirmed if the one-tailed test yielded p < .05. For this, linear regression and Wald tests were planned, using two dummy variables for the three groups compared and robust standard errors in Stata 15.1 (command *regress*). Inclusion criteria for the confirmatory analyses were: Answering *yes* to *the example describes a (fictional) dialogue between two scientists* and agreeing *weakly* or *very weakly* with *drinking plain water over years can only be investigated by observing, not by manipulating this behavioral variable*.

### Sampling

Sampling was based on the website scimagojr.com (https://www.scimagojr.com/journalrank.php?area = 3200, accessed on 9 August, 2021) that lists 1314 psychology journals. The journals were randomly ordered (using the *random* and *set seed* functions in Stata). A student was then instructed to do the following, journal by journal:

- Use the first issue of 2020.
- Consider the first 5 papers (as many as there are if < 5).
- For each of these papers, extract the corresponding author's email address. If the corresponding author's email address is not provided in a paper, search for it on the Internet. If the email address cannot be found, omit the author from the sample (e.g. a journal that should contribute 5 authors to the sample might contribute only 4).
- If an article has more than one corresponding author, take the first one mentioned.
- If a paper has no corresponding author mentioned, take the first author.
