# Supplementary material for: Avoidance of causality outside experiments: Hypotheses from cognitive dissonance reduction
Source: Sci Prog. 2024 Apr 3;107(2):00368504241235505. doi: 10.1177/00368504241235505 (PMC10993686; doi:10.1177/00368504241235505)
Supplement: sj-docx-1-sci-10.1177_00368504241235505 - Supplemental material for Avoidance of causality outside experiments: Hypotheses from cognitive dissonance reduction [file sj-docx-1-sci-10.1177_00368504241235505.docx]

**Appendix: methods used in the online study**

This appendix adds details on the pre-registered hypotheses and the sampling procedure. The project process was continuously recorded in the [OSF project](https://osf.io/msn9r). The record includes materials (the entire study process with all items used in LimeSurvey, version 5.3), Stata syntax for data processing and analysis, plans for exploratory analyses and their subsequent results, and open data.

**Pre-registered hypotheses and their analysis**

*Hypothesis 1:* The conflict group shows lower motivation than the control group.

*Hypothesis 2:* The benefits group shows higher motivation than the control group.

*Motivation* was defined as the mean of the items *I feel motivated to address causality outside experiments in my field* and “I feel motivated to dig into methods if necessary for this purpose”. A hypothesis was to be considered as confirmed if the one-tailed test yielded p < .05. For this, linear regression and Wald tests were planned, using two dummy variables for the three groups compared and robust standard errors in Stata 15.1 (command *regress*). Inclusion criteria for the confirmatory analyses were: Answering *yes* to *the example describes a (fictional) dialogue between two scientists* and agreeing *weakly* or *very weakly* with *drinking plain water over years can only be investigated by observing, not by manipulating this behavioral variable.*

**Sampling**

We add some details about the sampling procedure. Sampling was based on the website scimagojr.com (<https://www.scimagojr.com/journalrank.php?area=3200>, accessed on 9 August, 2021) that lists 1314 psychology journals. The journals were randomly ordered (using the *random* and *set seed* functions in Stata). A student was then instructed to do the following, journal by journal:

- Use the first issue of 2020
- Consider the first 5 papers (as many as there are if < 5)
- For each of these papers, extract the corresponding author's email address. If the corresponding author's email address is not provided in a paper, search for it on the Internet. If the email address cannot be found, omit the author from the sample (e.g. a journal that should contribute 5 authors to the sample might contribute only 4).
- If an article has more than one corresponding author, take the first one mentioned. If a paper has more than one corresponding author, take the first who is mentioned
- If a paper has no corresponding author mentioned, take the first author
