# Supplementary material for: Avoidance of causality outside experiments: Hypotheses from cognitive dissonance reduction
Source: Sci Prog. 2024 Apr 3;107(2):00368504241235505. doi: 10.1177/00368504241235505 (PMC10993686; doi:10.1177/00368504241235505)
Supplement: sj-pdf-2-sci-10.1177_00368504241235505 - Supplemental material for Avoidance of causality outside experiments: Hypotheses from cognitive dissonance reduction [file sj-pdf-2-sci-10.1177_00368504241235505.pdf]

Hello and welcome! Many thanks for your willingness to participate in this short follow-up. It will take only 5 minutes. The study has been approved by the ethics committee of the TU Dresden [SR-EK-370072021]

Section A: Consent and privacy statement

Hello and welcome! Many thanks for your willingness to participate also in this short follow-up. It will take only about 5 minutes. The study has been approved by the ethics committee of Dresden Technical University [SR-EK-370072021] First, we need to ensure that you agree with the consent and privacy and you feel able to confirm the following:

Declaration of consent for the processing of personal data in the context of the research project "Study on approaches to scientific questions" (Art. 6 para. 1 UAbs. 1 lit. a DSGVO)

Implementing institution: Dresden University of Technology Institute for Clinical Psychology and Psychotherapy Chair for Clinical Psychology and Behavioural Neuroscience

Alexander Giesche

Date of survey: from 03/2022

By endorsing "yes" below, I confirm ...

That I have read and understood the privacy policy and all questions have been satisfactorily answered to me in text or by email. That I am aware that my participation in the research project is voluntary. That I can revoke my consent at any time with effect for the future without having to give reasons. That I am capable of giving consent That I am voluntarily participating in the above-mentioned research project and consent to the processing of my personal data for the purposes described in the Data protection and privacy declaration That I am 18 years of age or older.

A1. I agree

Yes ☐

No ☐

A2. I have also read the informed consent letter and agree to its contents and to participate in this study.

Yes ☐

No ☐

A3. To merge the data from both surveys, we ask you to create a code. For the second survey, the code will be created again in the same way. The code will only be used to merge the data and will be deleted afterwards. [_] 2nd letter of your mother's first name[_] 2nd letter of your own first name[_ _] Day of your birth (two digits)[_] 1st letter of your father's first name. Example:Your mother's name is KarenYour name is Terayou were born on the 7thyour father's name is JohnThen your code is ae07J Please enter your own code:

Free text

Section B: Knowledge

B1. When estimating the causal effect of a factor on an outcome one should control for mediators.

knowl1_fu

No 0
Yes 1
Don't know 2

B2. When estimating the causal effect of a factor on an outcome one should adjust for common causes.

knowl2_fu

No 0
Yes 1
Don't know 2

B3. When estimating the causal effect of a factor on an outcome one should adjust for colliders.

knowl3_fu

No 0
Yes 1
Don't know 2

Section C: Research practices

C1. In general, what to you think about the following issues?

	Very Weakly/not at all	Weakly	Moderately	Strongly	Very strongly
Causal conclusions must only be made with experimental studies (randomized experiments or randomized clinical trials, RCTs).	1	2	3	4	5

viewpoints_1_fu

	Very Weakly/not at all	Weakly	Moderately	Strongly	Very strongly	viewpoints_*_fu * is ...:
I feel insecure in dealing with causality.	1	2	3	4	5	2
viewpoints_2_fu I fear to make wrong causal claims, therefore I only investigate associations if an experiment is impossible.	☐	☐	☐	☐	☐	3
In my own field investigating associations covers the core research questions.	☐	☐	☐	☐	☐	4
Everything that is necessary for a causal conclusion must be taken from the data.	☐	☐	☐	☐	☐	5
Conducting an experimental study protects against bias through a selective, non-representative sample.	☐	☐	☐	☐	☐	6
Conducting an experimental study protects against bias through common causes of a factor of interest and an outcome (confounding bias).	☐	☐	☐	☐	☐	7
When adjusting for confounders I use the same confounders than other authors in my field do.	☐	☐	☐	☐	☐	8
Causality is ultimately a matter of belief.	☐	☐	☐	☐	☐	9
A researcher‘s belief justifies a causal conclusion.	☐	☐	☐	☐	☐	10
A correlation most often indicates a causal effect.	☐	☐	☐	☐	☐	11
It is hard to address each and every aspect in a study including causality.	☐	☐	☐	☐	☐	12
I prefer to forward methodical issues like causality to a methodical expert/statistician.	☐	☐	☐	☐	☐	13
Avoiding explicit causal language (“causal effect”) in a paper on a non-experimental study makes readers more likely to draw a causal conclusion.	☐	☐	☐	☐	☐	14
Causality is ultimately a matter of definition.	☐	☐	☐	☐	☐	15
It is sufficient to mention the limitation that a causal conclusion can not be drawn.	☐	☐	☐	☐	☐	16
I fear that adjustment for confounders would let my finding of an effect diminish.	☐	☐	☐	☐	☐	17
	☐	☐	☐	☐	☐	

Section D: Research practices (cont.)

D1. In general, what to you think about the following issues?

	Very Weakly/not at all	Weakly	Moderately	Strongly	Very strongly	
viewpoints_18_fu I feel motivated to address causality outside experiments in my field.	1	2	3	4	5	18
viewpoints_19_fu I feel motivated to dig into methods if necessary for this purpose.	☐	☐	☐	☐	☐	19

outcome_fu = mean of viewpoints_18_fu , viewpoints_19_fu

Section E: Final

E1. **Thank you very much for your cooperation and answers so far. As a final question, we would like to ask you what your thoughts, beliefs and experiences on dealing with causality are now. Take your time to reflect and respond.**

feedback_fu

Section F: Good bye!

F1. **Thanks a lot for participating in this study. Your effort is very valuable to us. We know you are very busy and and very much appreciate the time to participating in this study. We kindly ask that you do NOT post online about the purpose of the study or discuss this study with others until the study is complete, so that other participants are not influenced by these comments.**

Feel free to note any comments about the study below. If you have any questions, comments, or concerns, please feel free to contact Alexander Giesche. Alexander.Giesche@tu-dresden.de

Section G: Good bye

G1. **Thank you.**

Thanks a lot for your participation! We appreciate your time and effort to having done so. Your cooperation is very valuable. We kindly ask you to maintain confidentiality about the contents so that the study can produce accurate results.
